# Atopic Dermatitis and the Atopic March: What Is New?

**DOI:** 10.1155/2011/279425

**Published:** 2011-09-13

**Authors:** Annalisa Patrizi, Alessandro Pileri, Federica Bellini, Beatrice Raone, Iria Neri, Giampaolo Ricci

**Affiliations:** ^1^Division of Dermatology, Department of Internal Medicine, Geriatrics and Nephrology, University of Bologna, Via Massarenti 1, 40138 Bologna, Italy; ^2^Department of Pediatrics, University of Bologna, Via Massarenti 9, 40138 Bologna, Italy

## Abstract

*Objective*. In this paper the authors review the management of atopic dermatitis (AD) and the association between AD and allergic respiratory diseases. *Data Sources*. PubMed databases, researching articles in the last 15 years. *Results*. Studies about atopic march are cross-sectional population studies at different ages. They show that the most important predisposing factor for atopy is a decrease of the filaggrin's expression. *Conclusions*. The most modern theories seem to show that the most important factor which starts the atopic march is represented by an impaired epidermal barrier. It causes an increase in skin permeability to allergens that could induce sensitization even in the airways. The major predisposing factor is a primary inherited epithelial barrier defect resulting from filaggrin gene mutation, but other factors may play a role in this complex mechanism. Further studies are needed to focus on AD treatment and preventive strategies.

## 1. Introduction

Atopic dermatitis (AD) is the commonest chronic cutaneous disease of childhood in the first years of life [1]. AD is often the first manifestation of allergic diseases. Literature data [2, 3] show a progression from AD to asthma: the so-called *atopic march*. In this paper we will focus reader's attention about the relationship between AD and respiratory allergic diseases.

## 2. History of AD

AD has a long history. It was firstly described in 1892 by Besnier [4] who named it “prurigo diathésique”. In order to highlight a possible association between the skin flare and genetic constitution, in 1927 Brocq suggested the term “constitutional eczema”. Wise and Sulzberger in 1933 changed this name into “atopic dermatitis”, finally adopted by Hanifin and Rajka in 1980 [5]. This is the current denomination adopted in the USA, while in Europe the most common definition is atopic eczema. In 2004 the World Allergy Organisation (WAO) Committee suggested to call “atopic eczema” any inflammatory condition determined by an IgE reaction, suggesting that the presence of eczema in atopic patients could be associated with or herald the development of some allergic diseases such as rhinitis and asthma [6].

## 3. Genetic State of Art

AD may be the first manifestation of allergic diseases and may be the first step of the so-called *atopic march.* A crucial role is played by filaggrin (filament-aggregating protein) that is involved in the epidermal barrier function. It is important for water permeability and for blocking the entry of microbes and allergens [7]. Filaggrin aggregates the keratin filaments and this process is essential for the formation of a normal stratum corneum and for the hydration of the skin. The filaggrin gene (FLG), located on chromosome 1q21 in the epidermal differentiation complex, encodes profilaggrin, a phosphorylated protein, precursor of filaggrin protein. Studies about FLG have underlined the link between early childhood eczema and the subsequent development of asthma. This is due to defective epidermal barrier function leading to increased allergen penetration and sensitization. Smith and coworkers in 2006 [8] demonstrated an association between ichthyosis vulgaris and a mutation in FLG, located on chromosome 1q21 (R501X and 2282del4). 

In their study 15 families have been studied; AD was present in 44% of individuals heterozygous for this gene and in 76% of homozygous individuals; none of the family member without mutation had AD. Actually it is thought that the frequency of null FLG mutations (5 in total) is 9% in the European population [9].

Further studies confirmed both filaggrin polymorphisms as a major risk factor for AD [10–15]. The International Study of Asthma and Allergies in Childhood showed that in a group of German children the presence of filaggrin variants increased more than 3 times the risk of developing AD and more than 2 times the risk of developing rhinitis regardless of the presence of AD [16]. 

Of course, ascertaining the precise contribution of FLG mutations to the overall prevalence of these atopic diseases is confounded by temporal and disease severity factors in conjunction with putative environmental effects.

AD and asthma present common immunological features, including elevated IgE levels, TH2 cytokines, lesional and peripheral eosinophilia, and common environmental triggers.

The hypothesis that asthma is secondary to allergic sensitization, occurring after epidermal skin barrier disruption, has been confirmed by the fact that asthma is found only in the subset of filaggrin mutation carriers with AD, however the precise mechanisms through which filaggrin mutations contribute to asthma and allergic rhinitis still remain unknown.

## 4. Epidemiology

Eczema is a very frequent skin disease. The prevalence of this condition ranges from 7% to 30% in children and from 2% to 10% in adults. In the last decades it has been increased with a percentage double or triple higher, in the developed countries [1].

In about 70–80% of patients, AD is associated with increased total IgE serum levels. Atopic diseases have different peaks of incidence at different ages during childhood. AD and food allergies have the highest incidence in the first 2 years of life. Sensitization to inhalant allergens is rare at that time of life. In later childhood, the prevalence of AD, food allergies, and food allergen sensitization decreases and the prevalence of asthma, allergic rhinitis, and sensitization to inhalant allergens rises [17]. 

Several studies have evaluated the association between AD and respiratory allergy, in particular Kulig et al. [18] have shown that, at the age of 5 years, 50% of children with AD have developed allergic respiratory diseases; Ricci et al. [19] have studied 252 children with AD, aged 6–36 months: the mean followup of these patients was 16.9 ± 2.9 years (range 13–22 years); in the first 10 years asthma appeared in 34.1% of cases and RC in 57.6%; they concluded that the severity and good control of AD was predicable for the onset of asthma. Ohshima et al. [20] in a 4-year follow-up study of 169 children with AD have shown that, at the end of the study, 35% of children developed asthma. van der Hulst et al. [21] in their systematic review have confirmed that young children with AD had a high risk of developing asthma in later childhood. 

According to a recent study by Spergel more than 50% of children with AD may develop asthma and approximately 75% allergic rhinitis during the first 6 years of life [3].

## 5. Clinical Features

In AD, typical lesions of acute eczema can be observed in a first time, then lichenification is prevalent due to the itching. Classically AD shows different clinical features in 3 different ages: first year of life (first step), childhood (second step), and adolescence/adulthood (third step). In the first step eczematous, exudating lesions mainly affect 3/5-month-old children. Lesions are located on the scalp and on the face where the central area is typically spared. In more severe patients the extensor surfaces of limbs are also involved. Parents usually refer to physician that their babies are becoming restless and they cannot sleep at night. In the second step flexural areas of arms and legs are commonly affected. Lesions are typical of subacute or chronic eczema, drier than those of the first step. Children frequently complain a bothersome itching. In the third step patients are adolescents or adult. Furthermore in this step they present lichenified eczema usually localized in one or a few skin areas such as the face, back of neck, hands, wrists, and antecubital and popliteal fossae. Itching is usually important. Inflammatory hyper pigmentation can be observed in periocular and neck areas and the last has been described as “dirty neck”. Similar lesions may be observed in other areas as on feet.

Diagnosis of AD is not so easy [5] because of the variety of clinical presentation, for this reason diagnostic criteria have been proposed. The first diagnostic criteria were proposed in 1977 by Hanifin and Lobitz [22]; in 1980 the diagnostic criteria by Hanifin and Rajka [5], were derived from Sulzberger's definition and from Hanifin and Lobitz's proposals. Furthermore Gu et al. refined these criteria in order to develop diagnostic guidelines for adults, children, and nonwhite ethnic groups suffering of AD [23]. 

In 1993, a consensus report of the European Task Force on Atopic Dermatitis [24] defined a validated scale, the SCORAD (SCORing Atopic Dermatitis), that considered the extent and severity of the eczematous lesions and the presence of two subjective symptoms: pruritus and loss of sleep. Then Oranje and coworkers [25], proposed a new SCORAD without patient's prospective, a patient-oriented SCORAD (PO-SCORAD) and the three-item severity (TIS) [26] score. The TIS score fits well with the more detailed objective SCORAD and can represent both as a prescreening system and as a useful tool for epidemiological studies. Recently in order to validate the PO-SCORAD index, a large European population study has been developed [27]. 

Hanifin et al. [28] proposed in 2001 another scoring system named EASI (Eczema Area and Severity Index), which can be applied to both AD children and adults. It considered four areas of the body: the head/neck, the trunk, and the upper and lower extremities. Each of the four body areas was separately assessed for erythema, induration/papulation/edema, excoriation, and lichenification. A score ranging from 0 to 3 was used to evaluate the severity of each sign in each of the four areas. In 2004, Charman et al. [29] published a new, validated score used both for adults and children called POEM (Patient-Oriented Eczema Measure), based on patients' assessments of what constitutes disease severity. It consists of questions about the frequency of seven symptoms: itch, sleep disturbance, skin bleeding, skin weeping/oozing, skin cracking, skin flaking, and skin dryness/roughness. 

IGA (Investigator Global Assessment) is another severity score tool that has been widely used in trials consisting of adults and children. IGA is a simple, 6-point scale, ranging from 0 (clear) to 5 (very severe disease), representing an overall evaluation of dermatitis that can be performed by the investigator at every consultation [30, 31].

A recent systematic review has identified 20 different published outcome measures, but the authors state that only EASI, SCORAD, and POEM have been adequately validated and recommend using EASI or SCORAD for an objective estimate of disease severity, plus the POEM as a measurement of eczema severity from the patient's perspective [32].

IGA is surely a good score but is not exclusive and specific to AD. Numerous differential diagnoses of AD should be considered: scabies, seborrhoeic dermatitis, Langerhans cell's histiocytosis mainly in childhood, and contact dermatitis in children and adults. 

## 6. Therapy

AD management is complex. Topical and systemic drugs are needed to control the disease. The American Academy of Dermatology has proposed detailed guidelines of care for eczema [33–35] based on the assessment of clinical severity by SCORAD or EASI indexes.

Recently, the PRACTALL (PRACTical ALLergy) consensus paper proposed a simple, step-based algorithm for eczema therapy depending on the severity [36]: (i) dry skin: it only needs hydration; (ii) mild, moderate, and severe eczema require, respectively, low-, mid-, and high-potency topical corticosteroids; furthermore topical calcineurin inhibitors TCIs can be helpful; (iii) systemic therapy is reserved to resistant eczema. This algorithm has some limitations: TCIs should not be used in children under 2 years and high-potency corticosteroids have to be avoided in children and in special sites such as the face. 

The use of TCIs has been debated, in the last few years. In 2005 [37], the Pediatric Advisory Committee of the Food and Drug Administration (FDA) labeled pimecrolimus and tacrolimus as dangerous, with a black-box warning about their potential carcinogenicity. Sporadic case of cancer set during treatment are reported. However, several groups [38–41] have focused on the evidence of TCIs' carcinogenetic role and have agreed that data are inconclusive and further studies must be done. Furthermore recent vehicle-controlled trials indicate efficacy of proactive treatment with tacrolimus in association with corticosteroides to prevent AE flares [42] At present however tacrolimus (Protopic) and pimecrolimus (Elidel) ointment have to be considered second-line treatments for AD in children [43].

## 7. AD and Quality of Life

AD is a serious problem for patients and their families, in fact, especially when severe, AD can be extremely disabling, causing psychological stress that, in young children, can involve the whole family. Clinicians should be aware of the psychological problems related to this disease and their impact on the child and parents' quality of life (QOL) [44].

### 7.1. AD and Family Life

In 1998, Lawson et al. [45] focused on QOL in the families of children affected by AD. They worked out the Dermatitis Family Impact Questionnaire, whose aim was to understand the family aspects most influenced by the disease. Their results showed that 74% of AD parents described a general burden of extra care, for example, relating to household cleaning and washing, preparing meals and shopping; 71% of parents described psychological pressures including feelings of guilt, exhaustion, frustration, resentment, and helplessness; night-time itching and scratching caused delay in getting the child to sleep and led to parental frustration and exhaustion in 64%. So for the 66% of families a “normal” family life was not possible. Furthermore Lawson et al. [45] reported that 63% of children with AD had current sleep problems and most had had sleep disturbance at some time. Patients with AD scratch more during sleep than patients with other chronic dermatologic diseases. Pauli-Pott et al. in their study [46] stated that mothers of children with AD were more helpless, depressed, and overprotective than mothers of healthy children. 

Ricci et al. [44] considered the parents of 45 children aged 3–84 months affected by AD were asked to complete two validated questionnaires after clinical examination (Infants' Dermatitis Quality of Life Index and Dermatitis Family Impact questionnaire). Children's QOL appeared slightly moderately altered (mean score 10.2) compared with the value of a control group (3.3), and itching, sleep problems, and the influence of the disease on the child's mood were the cause of greatest discomfort for the child. Family QOL appeared moderately altered (mean score 11) compared with the value of the control group (7.4). The greatest problem was the disturbed sleep of the family members. Other important problems were the economic cost for the management of the disease and the tiredness and irritability caused by the disease in parents.

## 8. Conclusions

AD is a complex disease with needs a well standardized clinical approach. Furthermore AD often preludes to other atopic diseases. Our hope is that, in the next future, dermatologists and paediatric allergists will have standardized guidelines based on international consensus conferences. Similarly to other chronic diseases such as allergic asthma, the use of step-based therapy could be useful in the management of the disease [4]. The evaluation of the quality of life of patients and their families represents a new target, with a global consideration of problems no more limited to the skin. Although the disease mainly affects childhood, in some cases it may persist even in adulthood and may be associated with asthma and/or allergic rhinitis. The percentage of AD persistent cases is extremely variable, ranging from 8–13% to 60–70% [1]. On the other hand, healing data reported in the literature widely vary: some authors report 50–70% healing at the age of 10 years, others [47] 43.2% at the age of 3, and some refer to a general improvement in AD severity in the first 5/7 years of life [19]. The relationship between asthma and AD is complex, because different and unknown mechanisms are involved in the atopic march. Further studies about loss of function in filaggrin gene are needed to elucidate the risks related to epidermal barrier defects. Potential targets for barrier repair and prevention of atopic diseases will play a crucial role in future therapy. Much data point towards a strong correlation between AD in early infancy and the subsequent appearance of asthma. The risk of developing asthma in children with AD is highly variable: according to some authors, the prevalence is 25%, while others suggest higher values up to 80% [17, 19]. In a recent study conducted by our team [1], the percentages of appearance of respiratory allergies is lower; in fact only 37.5% of children with AD have developed respiratory pathologies (rhinoconjunctivitis in 27% of cases and asthma alone in 17%) after a long-term followup. In this study the high efficacy reached in preventing respiratory allergies has been attributed to the good control of AD in the first years of life gained through the constant and effective collaboration between dermatologists and pediatric allergologists, sometimes with the aid of a psychologist.

##  Conflict of Interests

The authors declare no conflict of interests.
